# Correlation of clinical sepsis definitions with microbiological characteristics in patients admitted through a sepsis alert system; a prospective cohort study

**DOI:** 10.1186/s12941-022-00498-3

**Published:** 2022-02-22

**Authors:** David Yu, David Unger, Christian Unge, Åsa Parke, Jonas Sundén-Cullberg, Kristoffer Strålin, Volkan Özenci

**Affiliations:** 1 Division of Clinical Microbiology, Department of Laboratory Medicine, Karolinska Institutet, Stockholm, Sweden; 2 Trauma & Reparative Medicine Theme, Karolinska University Hospital, Stockholm, Sweden; 3 Department of Medicine Huddinge, Karolinska Institutet, Stockholm, Sweden; 4 Department of Clinical Microbiology, Karolinska University Hospital Stockholm, Stockholm, Sweden

**Keywords:** Sepsis, Blood culture, Focus of infection, Microorganisms

## Abstract

**Background:**

Sepsis was recently redefined as a life-threatening organ dysfunction caused by a dysregulated host response to infection. With this redefinition (Sepsis-3), clinical and microbiological characteristics of patients with sepsis may differ from the patients fulfilling the previous definition (Sepsis-2).

**Purpose:**

To describe differences in clinical and microbiological characteristics of sepsis episodes between Sepsis-3 and Sepsis-2. The secondary aim was to compare blood culture outcomes between episodes fulfilling Sepsis-3 and Sepsis-2 criteria, respectively.

**Methods:**

A prospective study design was used to include patients presenting with clinically suspected sepsis in the emergency department. Six blood culture bottles were collected from each patient. Blood cultures were described as having clinically relevant growth, contaminant growth, or no growth. Clinical and laboratory data were collected from medical records and the laboratory information system.

**Results:**

The analysis included 514 episodes. There were 357/514 (79.5%) Sepsis-3 and 411/514 (80.0%) Sepsis-2 episodes. In total, 341/514 (66.3%) episodes fulfilled both Sepsis-3 and Sepsis-2 criteria. Blood cultures were positive for clinically relevant growth in 130/357 (36.1%) and 145/411 (35.3%) episodes in Sepsis-3 and Sepsis-2, respectively. Other clinical and microbiological characteristics did not differ between Sepsis-3 and Sepsis-2.

**Conclusions:**

A high proportion of patients included through a sepsis alert system fulfilled both Sepsis-3 and Sepsis-2 criteria. The performance of blood cultures in detection of microorganisms was poor and were similar in Sepsis-3 and Sepsis-2 patients.

**Supplementary Information:**

The online version contains supplementary material available at 10.1186/s12941-022-00498-3.

## Introduction

### Background

Sepsis is defined as life-threatening organ dysfunction caused by a dysregulated host response to infection [1, 2]. The clinical definition of sepsis has varied over time, and the current definition was introduced in 2016 as Sepsis-3 [2, 3]. The major change was the use of parameters related to organ dysfunction as a consequence of infection, in contrast to the systemic inflammatory response syndrome (SIRS) used in the previous definition (Sepsis-2). Early optimal blood culture sampling is a cornerstone of sepsis management [4, 5], and blood culture is widely accepted as the gold standard for microbiological diagnostics in sepsis [6].

The microbiological findings in sepsis according to the previous definition have been well studied. In particular, the proportion of positive blood cultures in cases fulfilling Sepsis-2 criteria is reported to be around 30% in several studies [1, 7–9].

### Importance

In contrast to Sepsis-2, the microbiological characteristics in Sepsis-3 cases has scarcely been studied, two separate studies have reported the rate of blood culture positivity to be 22% and 48.2% [10, 11]. Sepsis treatment recommended by current guidelines relies on data originally from Sepsis-2 data [3]. However, it is possible that differences in the microbiological spectrum between Sepsis-3 and Sepsis-2 have an impact on the approach to the diagnosis and treatment of sepsis in future guidelines. Therefore, there is a need to characterize clinical and microbiological features in patients with Sepsis-3 and compare to those with Sepsis-2.

### Goals of this investigation

The primary aim of the study was to analyze clinical and microbiological characteristics in patients with suspected sepsis, according to both Sepsis-3 and Sepsis-2 definitions.

## Materials and methods

### Study design and setting

This prospective clinical study was performed at Karolinska University Hospital Huddinge, Stockholm, Sweden. The hospital has 760 beds and the ED had 53,508 visits in 2019 [12]. In the Emergency Department, all patients are routinely subjected to triage with the Rapid Emergency Triage and Treatment System [13] which is outlined in Additional file 1. As described in a previous study [14], the sepsis alert was triggered for triage signs of organ dysfunction combined with signs of infection, i.e. fever, history of fever, or clinical suspicion of infection (Additional file 2). Patients who triggered the sepsis alert were subjected to a multidisciplinary bedside assessment by physicians from the Emergency Department, the Department of Infectious Diseases and the Intensive Care Unit within 15 min, to optimize clinical assessment and treatment.

### Selection of participants

Consecutive adult patients who triggered the sepsis alert from September 2017 to February 2019 were included in the study. Patients with suspected sepsis accorded to the sepsis alert were included prospectively, thus patients with a final diagnosis other than sepsis and/or infection were also included in the study. A suspected sepsis episode, here referenced as an “episode,” was defined as a patient who triggered the sepsis alert system. Episodes were excluded if fewer than six blood culture bottles were collected, or if there was uncertainty that the collection of blood was performed in accordance with the study protocol. Only the first episode was included if the same patient triggered the sepsis alert system more than once during the study period.

### Blood culture collection and transportation

Three blood culture sets, each one consisting of an aerobic (BactAlert FA Plus) and an anaerobic (BactAlert FN Plus) blood culture bottle, were sampled from each patient who triggered the sepsis alert system. The blood culture bottles were transported to the Department of Clinical Microbiology, Karolinska University Hospital according to routine practice, and were analyzed according to the standard routine. During the initial period of this study, between September 2017 and September 20, 2018, the blood culture bottles were incubated in BacT/ALERT 3D (Bio-Merieux, France). Beginning on September 20, 2018, the clinical microbiology laboratory changed its blood culture system to BacT/ALERT Virtuo (Bio-Merieux, France). The BactAlert FA Plus and BactAlert FN Plus blood culture bottles were used throughout the study. Blood cultures were incubated in the system until they signaled positive or for a total of five days. In positive blood cultures, samples were Gram stained and then subcultured on agar plates. Identification of the microorganisms were later made using matrix-assisted laser desorption/ionization time-of-flight mass spectrometry (Bruker Daltonik, Bremen, Germany). Standard laboratory procedures were used for antibiotic susceptibility testing.

### Definition of clinically relevant growth and contaminant growth

Information regarding blood culture results in terms of isolate identification and time to detection was collected from the laboratory information system. Detected isolates were defined as clinically relevant growth or contaminant growth, according to an improved version of the methods used in previously published reports [14, 15]. Clinically relevant growth was defined as growth of pathogenic microorganisms in at least one blood culture bottle. Contamination was defined as such if two criteria (A and B) were fulfilled. First (A), isolates commonly regarded as contaminants (coagulase-negative staphylococci, *Corynebacterium* spp., *Macrococcus* spp., *Micrococcus* spp., and *Facklamia* spp.), as described by previous studies and guidelines, were considered to be potentially contaminant if they grew in three or fewer of the six bottles. Second (B), the potential contaminants had to show no growth in any other relevant microbiological sample (urine, skin/soft tissue, lower respiratory tract, cerebrospinal fluid, pleural/ascitic drainage) within ± 5 days of blood culture sampling.

### Data collection and analysis

Clinical and microbiological data were collected using the hospital´s electronic record system and the electronic laboratory information system, respectively. Sepsis-3 was considered to be present if two criteria (A and B) were fulfilled: A) infection was clinically suspected at admission to the ED; B) organ dysfunction corresponding to an increase in sequential organ failure assessment (SOFA) score ≥ 2 from baseline was present. Baseline SOFA score was determined by the best (i.e. most physiological) data points during a 3-month period before inclusion in the study. In case of missing baseline data, the score for that data parameter was assumed to be zero. In the present study, (A) was defined as present if broad spectrum antibiotics were initiated within 48 h after time of blood culture sampling and ongoing for at least 4 days afterwards as described in previous epidemiological studies [16].

Sepsis-2 was considered to be present if two criteria (A and C) were fulfilled. First (A), defined as above. Second (C), two or more SIRS criteria were present.

### Statistical analysis

Frequencies and percentages were used to summarize categorical variables, while means and standard deviations, together with medians and interquartile ranges, were used to summarize numerical variables. Statistical analyses were carried out using SPSS (IBM Corp. Released 2015. IBM SPSS Statistics for Windows, Version 23.0. Armonk, NY: IBM Corp.)

## Results

**Fig. 1:**
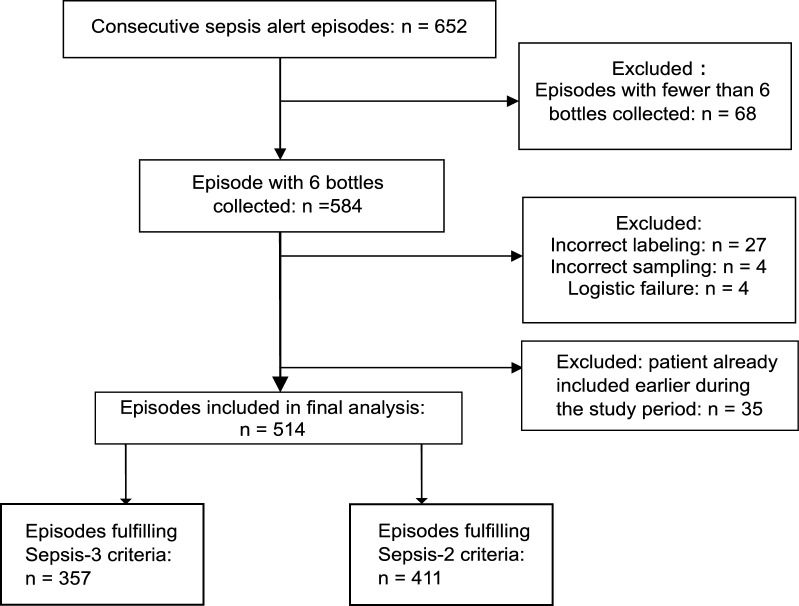
Flow chart of the study population

In total, 652 episodes triggered the sepsis alert system during the study period. Figure 1 depicts the study flow chart and reasons for exclusion. After exclusion 514 episodes were included in the final analysis. The mean patient age was 69.3 (SD ± 17.0) years and 60.5% were male. In total, 1542 blood culture sets (3084 blood culture bottles) were collected, each consisting of one BacT/Alert FA Plus and one FN Plus bottle.

### Characterization of study subjects

**Fig. 2:**
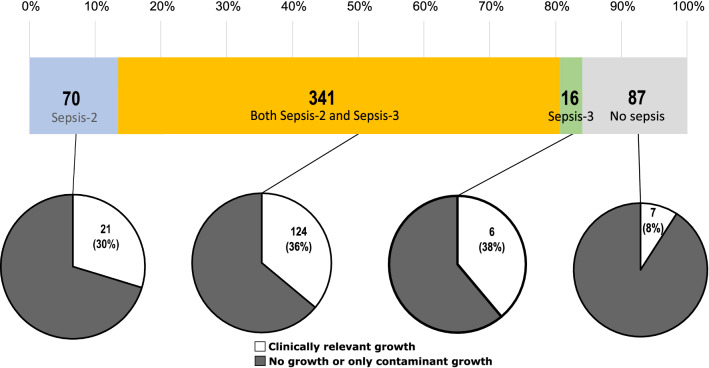
Clinical classification of suspected sepsis episodes and blood culture results. Numbers inside the horizontal bar denote episodes fulfilling criteria for clinical classification of Sepsis-3, Sepsis-2, both, or none. Percentages above the bar denotes the cumulative percentage of all included episodes. Numbers inside circles denote episodes with clinically relevant growth, and percentages in parenthesis denote percentage of all episodes in the associated clinical classification

Overall, 357/514 (69.5%) episodes fulfilled Sepsis-3 criteria (from here on called Sepsis-3 positive episodes) and 411/514 (80.0%) episodes fulfilled Sepsis-2 criteria (from here on called Sepsis-2 positive episodes). In total, 341/514 (66.3%) episodes fulfilled both Sepsis-3 and Sepsis-2 criteria. The number and proportion of episodes fulfilling either, both or none of the two studied criteria for sepsis (Sepsis-3 and Sepsis-2) is shown in Fig. 2. The clinical characteristics of episodes that fulfilled none, one or both sepsis definitions are described in detail in Table 1. Median (IQR) SOFA score was 3 (3–5) in the Sepsis-3 positive group, and 3 (2–5) in the Sepsis-2 positive group. 28-day mortality was 55/357 (15.4%) and 59/411 (14.4%) in the Sepsis-3 and Sepsis-2 groups, respectively.

**Table 1:** Clinical characteristics of the episodes (N = 514 episodes)

Characteristic	Sepsis-3 positive^a^/Sepsis-2 positive (n = 341)	Sepsis-3 negative/Sepsis-2 positive (n = 70)	Sepsis-3 positive/Sepsis-2 negative (n = 16)	Sepsis-3 negative/Sepsis-2 negative (n = 87)
Demographics				
Male—n (%)	209 (61.3)	42 (60.0)	10 (62.5)	50 (57.5)
Age—years^b^	73.2 (14.0)	60.2 (19.5)	71.6 (15.8)	60.8 (20.3)
28-day mortality n (%)	54 (15.8)	5 (7.1)	1 (6.3)	8 (9.2)
Admission to intensive care unit during hospital stay, n (%)	38 (11.1)	1 (1.4)	3 (18.8)	2 (2.3)
SOFA score^c^	3 (3–5)	1 (1–2)	3.5 (2–6)	2 (1–3)
Co-morbidities, n ()				
Congestive heart failure	66 (19.4)	8 (11.4)	4 (25.0)	9 (10.3)
Diabetes mellitus	101 (29.6)	15 (21.4)	4 (25.0)	32 (36.8)
Ischemic heart disease	50 (14.7)	5 (7.1)	2 (12.5)	7 (8.0)
Peripheral vascular disease	35 (10.3)	4 (5.7)	0 (0.0)	5 (5.7)
Cerebrovascular disease	74 (21.7)	9 (12.9)	3 (18.8)	12 (13.8)
Malignancy	86 (25.2)	9 (12.9)	5 (31.3)	14 (16.1)
Chronic kidney failure (GFR < 60)	98 (28.7)	8 (11.4)	4 (25.0)	16 (18.4)
Chronic pulmonary disease	52 (15.2)	18 (25.7)	2 (12.5)	10 (11.5)
Chronic liver failure	9 (2.6)	2 (2.9)	1 (6.3)	3 (3.4)
Source of infection^d^				
Respiratory tract	127 (37.2)	25 (35.7)	6 (37.5)	2 (2.3)
Urinary tract	80 (23.5)	15 (21.4)	4 (25.0)	0 (0.0)
Abdominal	27 (7.9)	5 (7.1)	0 (0.0)	2 (2.3)
Soft tissue/skin/skeletal/joint	26 (7.6)	10 (14.3)	2 (12.5)	0 (0.0)
Central nervous system	2 (0.6)	0 (0.0)	1 (6.3)	1 (1.1)
Endocarditis	5 (1.5)	0 (0.0)	0 (0.0)	0 (0.0)
Other/unknown	81 (23.8)	15 (21.4)	3 (18.8)	1 (1.1)
No suspected infection	N/A	N/A	N/A	81 (93.1)

*SOFA* Sequential Organ Failure Assessment, *GFR* glomerular filtration rate

^a^Sepsis-3 positive episodes were defined as present if there was an increase in SOFA score by 2 compared to baseline, as well as evidence of infection. Infection in this study was defined as present if the patient was administered intravenous antibiotic therapy within 48 h from admission and during at least 4 days. Sepsis-2 positive episodes were defined as present if there were 2 or more SIRS criteria in combination with infection

^b^Denotes mean ± standard deviation

^c^Denotes median (interquartile range)

^d^Two or more sources of infection were found in 8 episodes, so total percentage exceeds 100

### Microbiological findings

Growth in blood culture was noted in 193/514 (37.5%) episodes. Clinically relevant growth was found in 158 episodes (30.7%), of which 9 concomitantly had contaminant growth. A total of 35 episodes (6.8%) had only contaminant growth. Among 158 episodes with relevant growth, monomicrobial growth was detected in 132 cases (83.5%) and polymicrobial growth in 26 (16.5%). The microbiological findings in episodes that fulfilled none, one or both sepsis definition is described in detail in Table 2 and Fig. 2. When Sepsis-3 positive episodes were analyzed, 130/357 (36.4%) episodes had clinically relevant blood culture growth. For Sepsis-2 positive episodes, 145/411 (35.3%) episodes had clinically relevant blood culture growth. Contamination was found in 30/357 (8.4%) of Sepsis-3 positive episodes, and 35/411 (8.5%) of Sepsis-2 positive episodes. In patients who did not fulfill any of the sepsis definitions, 7/87 (8.0%) episodes had clinically relevant growth, and 8/87 (9.2%) episodes had contaminant growth. The clinically relevant growth in blood culture in relation to clinical sepsis diagnosis is depicted in Fig. 2.

**Table 2:** Microbiological characteristics of the episodes (N = 514 episodes)

Characteristic	Sepsis-3 positive^a^/Sepsis-2 positive (n = 341)	Sepsis-3 negative/Sepsis-2 positive (n = 70)	Sepsis-3 positive/Sepsis-2 negative (n = 16)	Sepsis-3 negative/Sepsis-2 negative (n = 87)
Microbiological findings, n (%)				
BSI episodes with clinically relevant growth	124 (36.4)	21 (30.0)	6 (37.5)	7 (8.0)
Contaminated episodes	29 (8.5)	6 (8.6)	1 (6.3)	8 (9.2)
Episodes with G+ isolates	57 (16.7)	7 (10.0)	4 (25.0)	2 (2.3)
Episodes with G− isolates	79 (23.2)	14 (20.0)	2 (12.5)	4 (4.6)
Fungal isolates	1 (0.3)	0 (0.0)	0 (0.0)	0 (0.0)
Episodes with anaerobic isolates	7 (2.1)	3 (4.3)	0 (0.0)	1 (1.1)
Time to detection (h)^b^	16.5 (11.6)	25.5 (22.4)	13.9 (6.0)	26.2 (13.5)

*BSI* blood stream infection

^a^Sepsis-3 positive episodes were defined as present if there was an increase in SOFA score by 2 compared to baseline, as well as evidence of infection. Infection in this study was defined as present if the patient was administered intravenous antibiotic therapy within 48 h from admission and during at least 4 days. Sepsis-2 positive episodes were defined as present if there were 2 or more SIRS criteria in combination with infection

^b^Denotes mean ± standard deviation

The rank order of clinically relevant and contaminant microorganisms is found in detail in Table 3. *Escherichia coli* was the most common microorganism in both Sepsis-3 positive as well as Sepsis-3 negative episodes. Overall, contaminant microorganisms in both Sepsis-3 positive and Sepsis-3 negative episodes consisted of coagulase negative staphylococci, 19/34 (55.9%) and 8/16 (50.0%) respectively.

**Table 3:** Rank order of microorganisms in sepsis-3 positive and sepsis-3 negative episodes

Sepsis-3^a^ positive episodes		Sepsis-3^b^ negative episodes	
Microorganism	n (%)	Microorganism	n (%)
**Clinically relevant microorganisms**			
*Escherichia coli*	43 (27.0)	*Escherichia coli*	10 (30.3)
*Staphylococcus aureus*	19 (11.9)	*Streptococcus pneumoniae*	2 (6.1)
*Klebsiella pneumoniae*	11 (6.9)	*Streptococcus anginosus (milleri)* group	2 (6.1)
*Staphylococcus epidermidis*	9 (5.7)	*Bacteroides fragilis*	2 (6.1)
*Pseudomonas aeruginosa*	8 (5.0)	*Parvimonas micra*	2 (6.1)
*Klebsiella oxytoca*	7 (4.4)	*Klebsiella pneumoniae*	1 (3.0)
*Enterococcus faecalis*	7 (4.4)	*Pseudomonas aeruginosa*	1 (3.0)
*Enterococcus faecium*	6 (3.8)	*Klebsiella oxytoca*	1 (3.0)
*Streptococcus agalactiae*	5 (3.1)	*Enterobacter cloacae*	1 (3.0)
Group A streptococci	5 (3.1)	*Moraxella (Branhamella) catarrhalis*	1 (3.0)
*Streptococcus pneumoniae*	4 (2.5)	*Acinetobacter* species	1 (3.0)
*Serratia marcescens*	3 (1.9)	*Campylobacter* species	1 (3.0)
*Proteus mirabilis*	2 (1.3)	*Haemophilus influenzae*	1 (3.0)
*Pantoea* species	2 (1.3)	*Staphylococcus aureus*	1 (3.0)
*Citrobacter freundii*	2 (1.3)	*Staphylococcus epidermidis*	1 (3.0)
*Streptococcus mitis* group	2 (1.3)	*Enterococcus faecalis*	1 (3.0)
Group B streptococci	2 (1.3)	*Enterococcus faecium*	1 (3.0)
Others^b^	22 (13.8)	Others^b^	3 (9.1)
**Total clinically relevant microorganisms**	159	**Total clinically relevant microorganisms**	33
**Contaminant microorganisms**			
Coagulase negative staphylococci	19 (55.9)	Coagulase negative staphylococci	8 (50.0)
*Staphylococcus epidermidis*	11 (32.4)	*Staphylococcus epidermidis*	6 (37.5)
*Corynebacterium* species	3 (8.8)	*Macrococcus* species	1 (6.3)
*Micrococcus luteus*	1 (2.9)	*Facklamia* species	1 (6.3)
**Total contaminant microorganisms**	34	**Total contaminant microorganisms**	16
**Total microorganisms**	193	**Total microorganisms**	49

^a^Sepsis-3 positive episodes were defined as present if there was an increase in SOFA score by 2 compared to baseline, as well as evidence of infection. Infection in this study was defined as present if the patient was administered intravenous antibiotic therapy within 48 h from admission and during at least 4 days

^b^*Stenotrophomonas maltophilia*, *Proteus vulgaris*, *Pseudomonas* species, *Enterococcus* species, *Globicatella* species, *Streptococcus mitis* group, *Lactobacillus* species, *Micrococcus luteus*, *Enterobacter cloacae*, *Enterococcus casseliflavus*, *Actinotignum schaalii*, *Helcococcus* spp., *Eggerthella lenta*, *Clostridium perfringens*, *Prevotella* species, unidentified gram negative anaerobe coccus, *Peptoniphilus* species, *Peptostreptococcus anaerobius*, *Candida parapsilosis*, *Micrococcus luteus* (all n = 1)

## Discussion

The present prospective study showed that most patients who triggered the sepsis alert in our emergency department fulfilled both Sepsis-3 and Sepsis-2 criteria, and the rate of blood culture positivity was similar but poor in both Sepsis-3 and Sepsis-2.

In the present study, episodes fulfilling Sepsis-3 criteria had a bacteremia rate of 36.4% and episodes fulfilling Sepsis-2 criteria had similar bacteremia rate of 35.3%. One previous study reports 22% bacteremia in patients with Sepsis-3 [10], and in another study, 48.4% of patients presenting with Sepsis-3 had positive blood cultures, however it was not stated if they consisted of only clinically relevant growth [11]. At least in the case of the higher reported rate of 48.2% only patients admitted to the ICU were studied, and is probably not representative of a Sepsis-3 population in the ED which also includes those who are not critically ill. In addition, previous data on patients with Sepsis-2 has shown bacteremia rates of around 30% [7–9, 17].

In the present study, the bacteremia rate was generally higher than previously reported. The reason for this discrepancy is unknown, however it might be related to differences in study design. In the present study, patients were included prospectively in the ED and there were no patients that received intravenous antibiotic treatment immediately prior to blood culture sampling. In addition, all included episodes had six blood culture bottles compared to four bottles that were used in the previous studies. As it is well known that blood culture yield correlates with sampled volume [6, 18, 19], this may have increased sensitivity by increasing the blood culture sample volume. We also employed a previously published method [14, 15] to define clinically relevant growth and contaminant growth. Notably, previous studies in both Sepsis-3 and Sepsis-2 patients included mostly retrospectively identified patients with sepsis [7] or did not specify whether contaminants were excluded in the blood culture positivity analysis [7, 9, 20].

The microbiological findings in Sepsis-3 and Sepsis-2 positive episodes were similar regarding both clinically relevant pathogens, contaminants, and microbiological spectrum. It is reasonable to suggest that the underlying reason for this might be the large overlap between the two groups in the present study. However, in the 86 episodes that had discrepant sepsis categorizations (Table 2), there were more episodes with clinically relevant growth and fewer contaminations in the Sepsis-3 positive group compared to the Sepsis-2 positive group. As the sample size of episodes with discrepant sepsis categorization was small, it was not possible to exclude a difference in blood culture results in this group.

The microorganisms implied in community acquired sepsis have previously been well studied, and the most common pathogens reported were *E. coli*, other *Enterobacterales* and *S. aureus* [8]. In the present study, the rank order of microorganisms in Sepsis-3 positive episodes is consistent with the findings in previous studies. However, in episodes that did not fulfill Sepsis-3 criteria, *Enterobacterales* other than *E. coli* as well as *S. aureus* were not as commonly isolated as in the Sepsis-3 positive episodes. It is possible that this difference reflects the importance of certain microbial virulence factors and interplay with immunological mechanisms as a major cause of organ dysfunction in sepsis [21].

There is a significant overlap between patients fulfilling Sepsis-3 and Sepsis-2 criteria. In the present results, 80% of patients fulfilling criteria for either sepsis criteria also fulfilled the other criteria. This contrasts to a recently published large study by Engoren et al. [22] which included 18,183 patients who had either Sepsis-3 or Sepsis-2, where only 6841 (37.6%) fulfilled criteria for both definitions. Additionally, our results contrast with another study by Todorovic et al. in which 24% of patients fulfilled both definitions of sepsis [23]. All patients included in the present study had a clinical suspicion of sepsis and were admitted to the ED at the time of inclusion. The discrepancy between the present results and previously published data is significant and may therefore be a result of patient selection criteria. In the previous study by Engoren et al., patients were screened retrospectively for sepsis, whereas in the present study, patients with suspected sepsis were included prospectively using a sepsis alert system. As trigger parameters for the sepsis alert system included components also present in SOFA (e.g. altered mental status, oxygen saturation) it is probable that the use of a sepsis alert system increased the likelihood that patients who fulfilled Sepsis-3 also fulfilled Sepsis-2 in our study.

The main strength of this study is its prospective design, using a clinical screening tool to select patients with suspected sepsis at presentation to the ED. The prospective design allowed for inclusion of patients with a “a priori” high likelihood of sepsis, regardless of the final diagnosis. The present results reflect the real-world scenario, where the final diagnosis is not available to the health care provider in the emergency department. Also, due to the prospective design, the blood culture sampling process was standardized, requiring six blood culture bottles to be sampled before initiation of antibiotic therapy in all patients included in the study.

The present study has several limitations. First, this was a single center study including only patients with a suspected infection upon presentation to the ED. This might introduce a bias in patient selection. However, the patients were carefully evaluated and represent a well-defined cohort for sepsis. Second, most patients included in the study had community acquired sepsis. However, the clinical criteria for Sepsis-3 and Sepsis-2 are general and applies for both community acquired sepsis and hospital acquired sepsis. Lastly, the present inclusion criteria used clinical triage criteria together with lactate measurements to include patients with suspected sepsis and it is possible that the criteria used in our study missed patients with sepsis. Existing sepsis screening tools have poor predictive values and current guidelines recommend against using a single screening tool. While we acknowledged that some patients with sepsis might have been missed, the aim of the current study was to include patients with a high probability of having sepsis.

## Conclusions

As the clinical definition of sepsis has changed, the performance of blood cultures and microbiological findings in sepsis may now be different. The present study showed that a high proportion of patients included through a sepsis alert system fulfilled both Sepsis-3 and Sepsis-2 criteria. The performance of blood cultures in detection of microorganisms was poor and were similar in Sepsis-3 and Sepsis-2 patients.

## Supplementary Information

**Additional file 1: Figure S1.** Criteria for initiation of the sepsis alert. Triage priority refers to RETTS priority (Fig. S2).

**Additional file 2: Figure S2.** RETTS triage system. GCS: Glasgow Coma Scale. RETTS: Rapid emergency triage and treatment scale.

## Data Availability

The datasets used and/or analysed during the current study are available from the corresponding author on reasonable request.

## Abbreviations

SIRS: Systemic inflammatory response syndrome
ED: Emergency department
SD: Standard deviation
IQR: Interquartile range
SOFA: Sequential organ failure assessment
GFR: Glomerular filtration rate
BSI: Blood stream infection
