# Supplementary material for: Correlation of clinical sepsis definitions with microbiological characteristics in patients admitted through a sepsis alert system; a prospective cohort study
Source: Ann Clin Microbiol Antimicrob. 2022 Feb 22;21:7. doi: 10.1186/s12941-022-00498-3 (PMC8864844; doi:10.1186/s12941-022-00498-3)
Supplement: Supplementary file 1 — Additional file 1: Figure S1. Criteria for initiation of the sepsis alert. Triage priority refers to RETTS priority (Fig. S2). [file 12941_2022_498_MOESM1_ESM.docx]

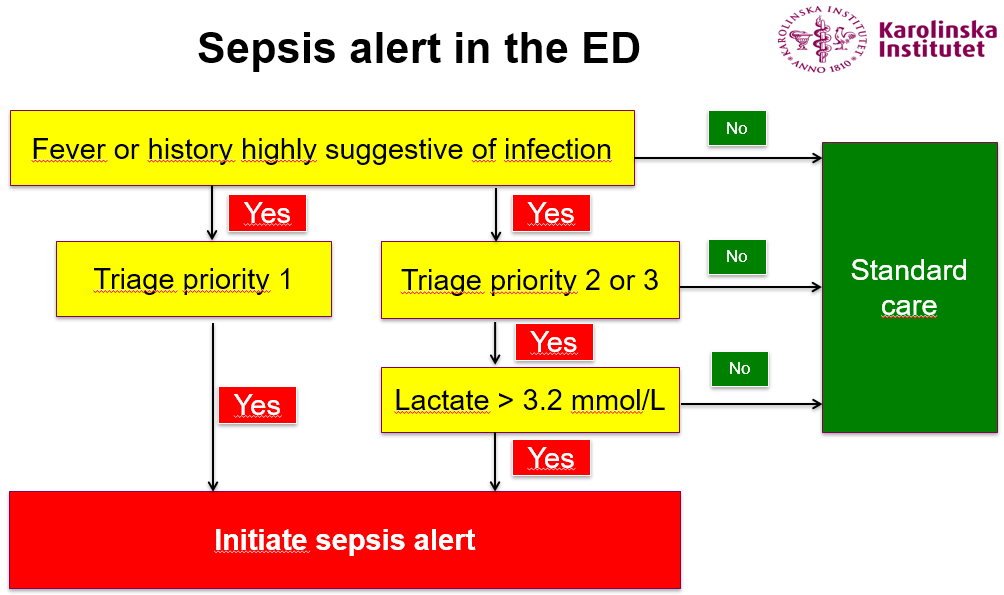


Additional file 1: Criteria for initiation of the sepsis alert. Triage priority refers to RETTS priority (Fig. S2)
