# Supplementary material for: Correlation of clinical sepsis definitions with microbiological characteristics in patients admitted through a sepsis alert system; a prospective cohort study
Source: Ann Clin Microbiol Antimicrob. 2022 Feb 22;21:7. doi: 10.1186/s12941-022-00498-3 (PMC8864844; doi:10.1186/s12941-022-00498-3)
Supplement: Supplementary file 2 — Additional file 2: Figure S2. RETTS triage system. GCS: Glasgow Coma Scale. RETTS: Rapid emergency triage and treatment scale. [file 12941_2022_498_MOESM2_ESM.docx]

**Triage priority 1** – Unstable patient, immediate assessment

- Obstructed airway
- Sat < 90% with O2 treatment
- Respiratory rate > 30 or < 8
- Heart rate > 130 if regular, > 150 if irregular
- Systolic blood pressure < 90 mmHg
- GCS < 8 or ongoing seizures

**Triage priority 2** – Unstable patient, with potentially ongoing life threat, emergent assessment (within 15 min)

- Sat < 90% on room air
- Respiratory rate > 25
- Heart rate > 120 or < 40
- GCS 8-12
- Temperature > 41°C or <35°C

**Triage priority 4** – stable patient, without ongoing life threat but in need of care within reasonable time

- Sat > 95% on room air
- Heart rate 50 - 110
- Alert
- Temperature 35 - 38.5°C

**Triage priority 3** – stable patient without ongoing life threat but in need of emergency care

- Sat 90-95% on room air
- Heart rate > 110 or < 50
- Acute confusion
- Temperature > 38.5°C

**Additional file 2:** RETTS triage system. GCS: Glasgow Coma Scale. RETTS: Rapid emergency triage and treatment scale.
